# Gas-Generating Photocatalytic Agents for Bacterial Infection Treatment

**DOI:** 10.34133/research.0672

**Published:** 2025-04-16

**Authors:** Yanling Hu, Kaiqi Yang, Ning Li, Dongliang Yang, Heng Dong

**Affiliations:** ^1^ College of Life and Health, Nanjing Polytechnic Institute, Nanjing 210048, China.; ^2^ College of Material Engineering, Fujian Agriculture and Forestry University, Fuzhou 350108, China.; ^3^ Fujian Key Laboratory of Drug Target Discovery and Structural and Functional Research, School of Pharmacy, Fujian Medical University, Fuzhou 350122, China.; ^4^ State Key Laboratory of Flexible Electronics (LoFE) & Institute of Advanced Materials (IAM), School of Physical and Mathematical Sciences, Nanjing Tech University (NanjingTech), Nanjing 211816, China.; ^5^ Nanjing Stomatological Hospital, Affiliated Hospital of Medical School, Institute of Stomatology, Nanjing University, Nanjing, Jiangsu 210008, China.

## Abstract

Bacterial infections markedly strain healthcare systems financially, compounded by the rise of drug-resistant strains and biofilm-associated infections. Gas therapy has emerged as a notable solution, disrupting biofilms and targeting resistant bacteria through controlled gas release mechanisms. However, achieving precise and controlled gas release remains a critical challenge for the successful implementation of gas therapy. In this perspective, we summarize recent advancements in photocatalytic gas release for treating bacterial infections. It also outlines crucial challenges that must be addressed to fully leverage this promising therapeutic strategy, enhancing its precision and effectiveness in clinical settings.

## Introduction

The emergence of antibiotic-resistant bacteria has emerged as a formidable challenge to global public health, leading to a persistent rise in intractable infections [1]. The proliferation of multidrug-resistant pathogens not only markedly elevates infection-related morbidity and mortality rates but also severely compromises clinical outcomes in vulnerable populations [2]. Furthermore, the formation of bacterial biofilms exacerbates this predicament [3]. These biofilms are intricately linked to the recurrence of chronic bacterial infections [4]. Within biofilms, bacteria construct robust protective barriers by secreting extracellular polymeric substances, effectively shielding themselves from both host immune defenses and conventional antimicrobial agents [5–7]. This unique protective architecture complicates the treatment of various infection types. Antibiotics are currently fundamental in treating bacterial infections, targeting vital processes such as cell wall synthesis as well as DNA, RNA, and protein synthesis [8]. Yet, managing drug-resistant or biofilm-associated infections often requires repeated high-dose antibiotics and surgical debridement [9]. This regimen extends treatment duration, increases costs, and amplifies the risk of adverse effects and unpredictable outcomes [10].

In recent years, gas therapy using molecules such as hydrogen, nitric oxide, carbon monoxide, and hydrogen sulfide has gained substantial attention as an innovative antibacterial strategy, particularly effective due to its ability to penetrate bacterial biofilms and exert antimicrobial effects directly within them [11,12]. Unlike traditional drugs, gas molecules are less prone to inducing resistance and offer enhanced tissue permeability, enhancing their potential for treating bacterial infections [13]. Moreover, gases that exhibit additional biological activities—such as anti-inflammatory, immune-regulating, and revascularization properties—can improve the wound healing process [14]. However, the clinical application of gas therapy is challenged by issues like uncontrolled release and potential side effects [12]. Photocatalysis technology has seen extensive application across various sectors, including environmental cleanup and solar energy conversion [15]. Recently, photocatalytic antimicrobial strategies have emerged as a green and sustainable alternative strategy for combating drug-resistant bacteria [16]. This technology leverages light irradiation to activate semiconductor photocatalysts, producing reactive oxygen species (ROS) that achieve potent antimicrobial effects [17]. Photocatalytic methods are advantageous over traditional antibiotics due to their cost-effectiveness, broad-spectrum activity, and reduced likelihood of inducing resistance [16]. The integration of photocatalytic and gas therapies exhibits a synergetic/enhanced antibacterial efficacy by enabling precise control of gas generation through light-regulated photocatalytic processes, presenting new avenues for bacterial infection treatment (Figure and Table). This perspective reviews the latest developments and challenges in merging photocatalytic and gas antimicrobial therapies, highlighting their promising roles in advancing infection treatment.

**Figure.**
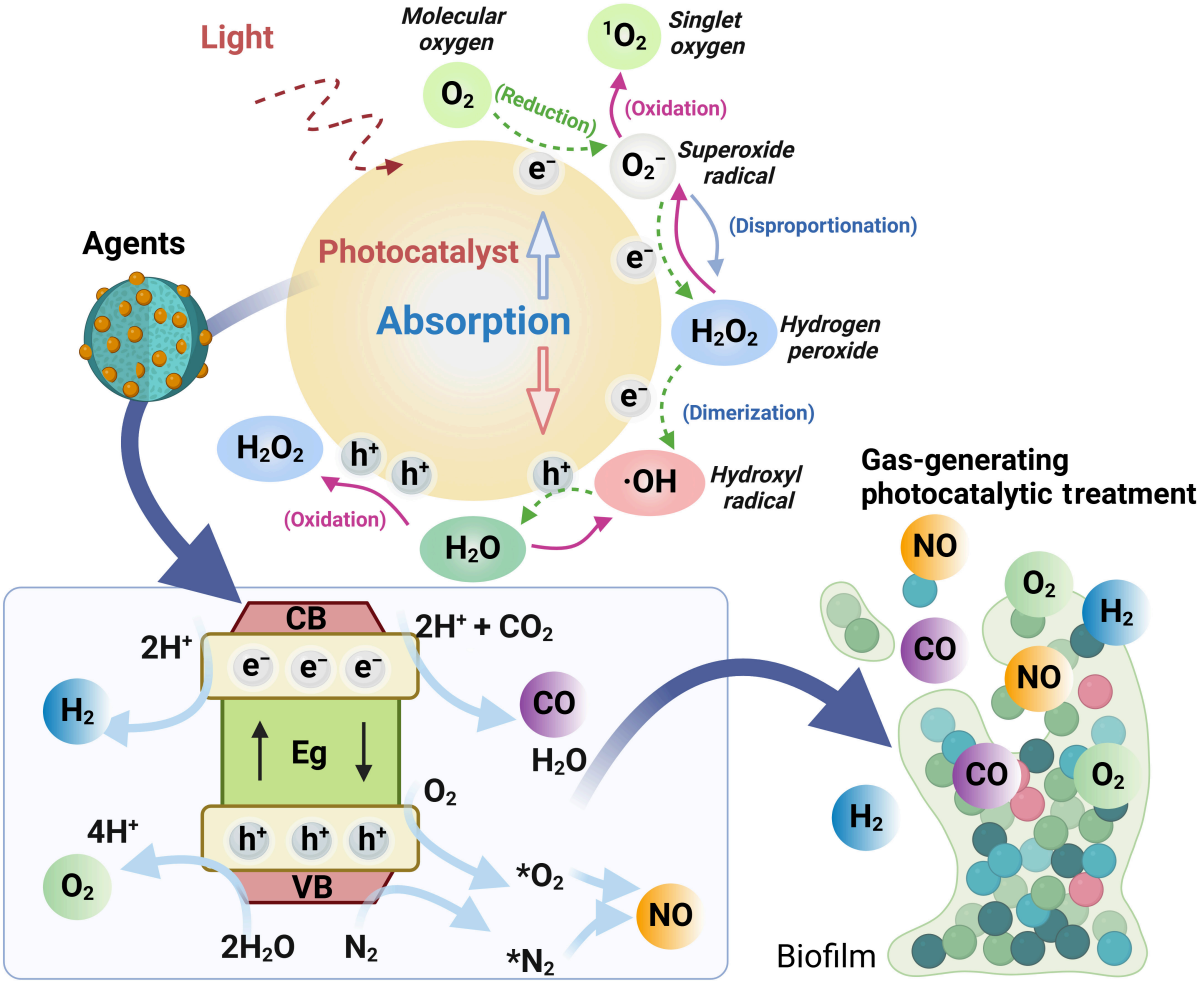
Photocatalytic agents with gas-generating capabilities for the treatment of bacterial infections. Reproduced from Ref. [16] with permission from American Chemical Society, copyright 2023. CB, conduction band; VB, valence band; Eg, bandgap energy.

**Table.** The biological function, antibacterial mechanism, and enhanced antibacterial mechanism of gas therapy

Gas	Physiological functions	Antibacterial mechanism	Synergistic/enhanced mechanism
H_2_	Antibacterial, antiapoptotic, anti-inflammatory, and antioxidant properties; promotion of wound healing by enhancing epidermal stem cell proliferation and extracellular matrix deposition	Induction of bacterial membrane rupture, disruption of intracellular oxidative stress balance	Amplification of ROS action by interfering with intracellular oxidative stress balance
NO	Antimicrobial activity, bacterial biofilm dispersion, vasodilation and blood pressure regulation, immune regulation, promotion of wound healing by revascularization and collagen deposition	Oxidative damage to biomolecules, induction of biofilm dispersion	Reaction with ROS to generate reactive nitrogen species
CO	Antimicrobial activity, anti-inflammatory	Inhibition of cellular respiration	Enhancement of the sensitivity of bacteria or bacterial biofilms to antimicrobial agents
O_2_	Involvement in the biological process of cellular respiration, energy production, immune defense, wound healing, and tissue repair	Enhancement of the sensitivity of bacteria to antibacterial agents	Immune cells need oxygen to produce a large number of ROS through the respiratory burst pathway, equipping them with potent anti-infection capabilities; promotion of the occurrence of oxygen-dependent type II photodynamics

ROS, reactive oxygen species.

## Photocatalytic Hydrogen Generation

Hydrogen (H_2_), a potent reductant in biological systems, exhibits remarkable antiapoptotic, anti-inflammatory, and antioxidant properties [12]. Recent studies have revealed that H_2_ also functions as an effective antibacterial agent, capable of inducing bacterial membrane rupture, disrupting intracellular oxidative stress balance, causing DNA damage, and impairing bacterial energy metabolism, ultimately leading to bacterial death [18,19]. Furthermore, H_2_ has been shown to enhance epidermal stem cell proliferation, promote extracellular matrix deposition, and induce macrophage polarization toward the M2 phenotype, thereby accelerating wound healing [20–22]. To achieve a controlled release of H_2_, several photocatalysts with both antibacterial activity and H_2_ production capabilities, such as Ag/Ni–BaWO_4_ and W_18_O_49_/g-C_3_N_4_, have been reported [23,24]. However, research on photocatalysis-controlled H_2_ release for antibacterial therapy remains in the nascent stages. Given the ability of H_2_ to disrupt bacterial redox balance, its integration with photocatalysis-mediated antibacterial strategies could significantly amplify the overall antibacterial efficacy. Additionally, leveraging hydrogen’s anti-inflammatory properties, we believe that photocatalysis-controlled H_2_ therapy holds immense potential for treating infected wounds characterized by excessive inflammatory responses.

## Photocatalytic Nitric Oxide Generation

Nitric oxide (NO), the most extensively studied endogenous gas, plays a pivotal role in protecting the body against exogenous pathogens. At high concentrations, NO induces oxidative damage to biomolecules, leading to bacterial inactivation [25]. Moreover, when NO gas therapy is combined with photocatalytic antibacterial strategies, NO reacts with ROS to generate reactive nitrogen species, which exhibits an even higher antibacterial activity, thereby significantly enhancing the therapeutic efficacy against bacterial infections. In addition, NO also plays an important role in cardiovascular health, immune regulation, wound healing, and bacterial biofilm dispersion. Currently, some photocatalytic agents have demonstrated the ability to catalyze the production of nitric oxide (NO) under mild conditions [26–28]. For instance, Kandoth et al. developed a novel ternary heterojunction photocatalytic material (CBB/TiO_2_/RuPS) by synthesizing Cs_3_Bi_2_Br_9_ (CBB) perovskite (PeV)/TiO_2_ core–shell structures and further loading [Ru(2,2′-bpy)_2_(4,4′-dicarboxy-2,2′-bpy)]^2+^ (2,2′-bpy, 2,2′-bipyridyl) (RuPS) onto its surface. CBB/TiO_2_/RuPS can generate NO, hydroxyl radicals, superoxide anions, and singlet oxygen under natural light exposure. The produced NO and ROS interact to form reactive nitrogen species, which markedly enhances antibacterial activity. In vitro antimicrobial tests demonstrated that CBB/TiO_2_/RuPS effectively eradicated *Campylobacter jejuni* and methicillin-resistant *Staphylococcus aureus*, as well as their associated biofilms [29]. However, the efficacy of the CBB/TiO_2_/RuPS photocatalytic agent in treating bacterial infections in vivo remains unverified. In follow-up work, the team synthesized NTFA@PeV@BA-PTZ nanocrystals (NCs), a novel photocatalytic material capable of releasing NO, by modifying the surface of CH_3_NH_3_PbBr_3_ PeV NCs with the NO donor 4-nitro-3-(trifluoromethyl)anilinium hydrobromide (NTFA) and the hole transport carrier phenothiazine-benzoic acid (BA-PTZ) [30]. Under sunlight irradiation, NTFA@PeV@BA-PTZ NCs demonstrated the ability to transfer holes from BA-PTZ, generating hydroxyl radicals through a photocatalytic process even under oxygen-deficient conditions. The exciton separation in PeV facilitates and accelerates the photoelectron transfer process of NTFA, enabling precise control of NO release. By leveraging exciton dissociation kinetics to achieve controlled generation of ROS and NO, the material effectively eliminated over 90% of methicillin-resistant *S. aureus* and *Escherichia coli* within biofilms. In a mouse wound infection model, NTFA@PeV@BA-PTZ NCs accelerated wound healing when activated by visible light, demonstrating its potential for in vivo therapeutic applications. These innovative approaches highlight the potential of combining NO gas therapy with photocatalytic materials for advanced antibacterial applications.

## Photocatalytic Carbon Monoxide Generation

Carbon monoxide (CO), as a gaseous signaling molecule, has demonstrated substantial therapeutic potential in disease treatment due to its antibacterial and anti-inflammatory properties. At high concentrations, CO can bind to terminal oxidase in bacteria, competing with oxygen and inhibiting cellular respiration, thereby effectively killing bacteria. Within the range of 10 to 500 ppm [31], CO demonstrates obvious anti-inflammatory activity, as evidenced by in vitro evaluations using macrophage models. In addition, macrophage-derived CO enhanced the activity of macrophage to clear pathogenic bacteria by activating NACHT-LRR-PYD-containing protein 3 (NALP3) inflammasomes [32]. Compared to ROS, CO has a longer half-life (approximately 3 to 7 h), enabling its effective penetration into bacterial biofilms. This property facilitates the disruption of deep-seated bacterial biofilms and enhances their susceptibility to ROS-based therapeutic strategies. Therefore, achieving controlled release of CO is crucial for harnessing its different therapeutic effects. Recently, advancements in photocatalytic carbon dioxide (CO_2_) reduction have enabled precise and controlled CO generation in vivo [33]. For example, Zhuang’s team [34] developed an Nb_2_C MXene-based photocatalytic nanoplatform (NNBC) for the treatment of bacteria-infected osteomyelitis. To enhance the photocatalytic CO production efficiency of Nb_2_C, nickel (Ni) nanoparticles were first loaded onto its surface. Subsequently, dopamine-functionalized amino-poly(ethylene glycol) was introduced into the surface of Ni/Nb_2_C through electrostatic interactions. Finally, a CO_2_ donor was incorporated into the Ni/Nb_2_C nanosheets via coordination between the 3,4-dihydroxyl groups on dopamine, iron ions, and bicarbonate. Under 1,064-nm laser irradiation, the photothermal effect of Nb_2_C triggers the degradation of carbonate, releasing CO_2_, which is then captured by Ni/Nb_2_C and reduced to CO. In vitro experiments demonstrated that NNBC, combining photothermal and CO-mediated antibacterial effects, achieved over 97% elimination rates for both *E. coli* and *S. aureus*. Additionally, NNBC markedly reduced the inflammatory response in macrophages. In a mouse model of osteomyelitis, NNBC markedly decreased pathogenic bacterial burden at the infection site and effectively mitigated excessive inflammatory responses, further promoting the regeneration of damaged tissue.

In addition to inorganic composites, Wu et al. [35] recently developed a single-component organic conjugated microporous polymer capable of directly reducing CO_2_ from the air to CO under light irradiation, simultaneously producing hydrogen peroxide (H_2_O_2_). The yields of CO and H_2_O_2_ reached impressive levels of 361.2 and 552.7 μmol h^−1^ g^−1^, respectively, demonstrating the polymer’s potential for use in photocatalytic-mediated CO gas therapy.

## Photocatalytic Oxygen Generation

Diabetic wounds are often characterized by a hypoxic (low-oxygen) environment, which delays wound healing. This condition arises primarily because the hyperglycemic environment in individuals with diabetes impairs angiogenesis, further compromising blood supply to the wound site and disturbing blood circulation. Poor circulation reduces the ability of blood to reach the wound site effectively, depriving the essential oxygen and nutrients [36]. Oxygen is an essential nutrient for wound healing (e.g., revascularization and tissue remodeling) and warding off infections [37]. In the face of wound infection, immune cells need oxygen to produce a large number of ROS through the respiratory burst pathway, equipping them with potent anti-infection capabilities. Consequently, there is an urgent need to develop advanced wound dressings with blood glucose regulation, oxygen generation, and antibacterial properties to effectively treat diabetic wound infections. To achieve that target, Sun et al. [38] engineered a multifunctional polyglutamic acid-based hydrogel using ethylene glycol diglycidyl ether as a cross-linking agent. The hydrogel’s versatility is achieved through the incorporation of glucose oxidase, tungsten oxide, and polydopamine. In diabetic-infected wounds, glucose oxidase catalyzes the conversion of wound glucose into gluconic acid and H_2_O_2_. Then, under 808-nm laser irradiation, the photocatalytic activity of tungsten oxide is activated, decomposing H_2_O_2_ into oxygen to ameliorate wound hypoxia. This cascade catalytic reaction enables simultaneous blood glucose regulation and oxygen synthesis at the wound site. Additionally, under 808-nm laser exposure, the polydopamine within the hydrogel generates localized hyperthermia, effectively killing pathogenic bacteria. When the laser is turned off, polydopamine’s antioxidant properties help scavenge excess ROS at the wound site, mitigating excessive inflammatory responses and promoting the healing of diabetic wounds. This multifaceted mechanism not only regulates blood glucose levels but also addresses hypoxia and infection in diabetic wound management.

Even though photocatalysis-controlled gas therapy demonstrates superior therapeutic efficacy in combating infections, several challenges remain that must be addressed. First, while both photocatalysis and gas therapy have shown broad antibacterial potential, their specific mechanisms of action require deeper investigation to fully harness their capabilities in anti-infective applications. Extensive research has been conducted on the antibacterial effects of photocatalysis, yet the potential impact on normal tissues remains underexplored. Additionally, gas molecules often function as physiological signaling molecules, regulating various biological processes, which complicates the clinical translation of gas therapy. Second, the antibacterial efficacy of gas therapy is closely linked to gas production performance. Current research relies predominantly on in vitro experiments to quantify gas generation; however, effective methods for monitoring gas release in vivo are still lacking. Moreover, there is a lack of standardization concerning the minimum dose required for antibacterial activity in both in vitro and in vivo settings. This absence of standardization complicates efforts to balance strong antibacterial effects with minimizing adverse effects on normal tissues [39]. Third, a systematic evaluation of the biosafety of photocatalytic materials is essential for their future clinical application. Beyond catalytic performance, the physiological effects of these materials—including cytotoxicity, neurotoxicity, and reproductive toxicity—must be thoroughly assessed [40]. Fourth, another area of concern is whether the production of therapeutic gases interferes with the photocatalytic process. Finally, compared to other exogenous stimulators such as ultrasound, magnetic fields, microwaves, and x-rays, the tissue penetration depth of light is relatively limited, posing a challenge for treating deep tissue infections. Fortunately, however, it has been found that near-infrared light exhibits greater tissue penetration than visible light, opening new possibilities for photocatalysis-mediated gas therapy [41]. Despite these challenges, near-infrared II light-triggered photocatalysis-controlled gas therapy shows strong clinical potential for fighting bacterial infections.
